# Pulmonary Aspergillosis in an Infant with Multiple Hepatic Hemangiomas

**DOI:** 10.3390/children13040556

**Published:** 2026-04-16

**Authors:** Zuzanna Karp, Jakub Czarny, Katarzyna Adamczewska-Wawrzynowicz, Alicja Bartkowska-Śniatkowska, Katarzyna Jończyk-Potoczna, Katarzyna Derwich

**Affiliations:** 1 Student Scientific Society, Poznan University of Medical Sciences, 61-701 Poznan, Poland; 86916@student.ump.edu.pl (Z.K.); 86156@student.ump.edu.pl (J.C.); 2 Department of Pediatric Oncology, Hematology and Transplantology, Institute of Pediatrics, Poznan University of Medical Sciences, 60-572 Poznan, Poland; kadamczewska-wawrzynowicz@ump.edu.pl; 3 Department of Pediatric Anesthesiology and Intensive Therapy, Institute of Pediatrics, Poznan University of Medical Sciences, 60-572 Poznan, Poland; asniatko@ump.edu.pl; 4 Department of Pediatric Radiology, Institute of Pediatrics, Poznan University of Medical Sciences, 60-572 Poznan, Poland; jonczyk@ump.edu.pl

**Keywords:** infantile hepatic hemangioma, pulmonary aspergillosis, immunosuppressive therapy, antifungal therapy

## Abstract

**Highlights:**

Infantile hepatic hemangiomas (IHH) in childhood are associated with numerous complications, and prognosis is often unfavorable.

**What are the main findings?**

- This case highlights the complexity of IHH with recurrent severe respiratory and pulmonary infections, complicated by a cavitary lesion with necrotic tissue due to aspergillosis and airway compression.
- The treatment regimen with corticosteroids and sirolimus contributed to immunosuppression, and the substantial hepatomegaly worsened respiratory function and predisposed the patient to infections.

**What are the implications of the main findings?**

- The goal of IHH therapy is to prevent life-threatening complications.
- Such complex cases require careful monitoring and individualized treatment.

**Abstract:**

**Background**: Infantile hepatic hemangiomas (IHH) are common benign vascular tumors in infancy with diverse presentations. **Methods**: We report a 7-week-old infant presenting with hepatosplenomegaly, multiple skin and hepatic hemangiomas, anemia, and recurrent lung infections. **Results**: Treatment included propranolol, corticosteroids, and sirolimus, along with antifungal prophylaxis with fluconazole. The patient developed pneumothorax and pulmonary aspergillosis. Despite antifungal therapy with voriconazole and liposomal amphotericin B, along with surgical intervention, her condition deteriorated, resulting in multi-organ failure and death at 8.5 months of age. **Conclusions**: This case illustrates the complexity of IHH management and highlights the risk of severe infections during immunosuppressive therapy even when standard prophylaxis protocols are applied.

## 1. Introduction

Infantile hemangiomas (IH), including infantile hepatic hemangiomas (IHH), are the most common benign vascular tumors in childhood. IHH affects approximately 4.5% of term neonates [1]. Low birth weight, prematurity, and a family history are among the identified risk factors for IHH [2]. Most of these tumors regress spontaneously. However, some forms are associated with numerous complications, and even with treatment, the prognosis is fairly unfavorable. Cutaneous hemangiomas often accompany hepatic infantile hemangiomas in infants.

IHH can be divided into three subtypes: diffuse hepatic hemangiomas (DHH), focal hepatic hemangiomas, and multifocal hepatic hemangiomas, which may progress to DHH [2]. DHH is characterized by almost complete displacement of the liver parenchyma and can cause severe complications, such as multisystem organ failure, compression of surrounding organs, vasculature, hypothyroidism, bleeding, and abdominal compartment syndrome [3,4,5].

The goal of IHH therapy is to prevent life-threatening complications while avoiding the negative consequences of advanced treatment. Propranolol is the first-line treatment for patients with IHH. In severe cases, corticosteroids or sirolimus may be considered [6].

We report a case of a 7-week-old infant who presented with IHH, progressing to cardiac and respiratory arrest, as well as recurring severe lung infections, even when standard prophylaxis protocols are applied.

## 2. Case Report

**Figure 1:**
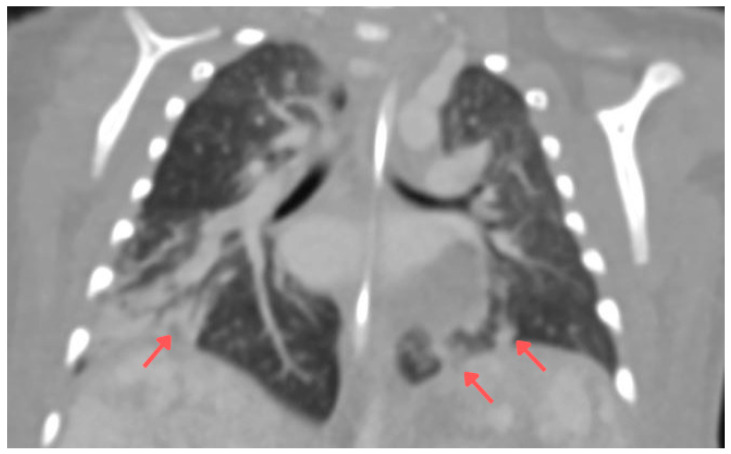
Contrast-enhanced chest CT (coronal section) performed during the first week of hospitalization. Some lesions were marked with arrows.

**Figure 2:**
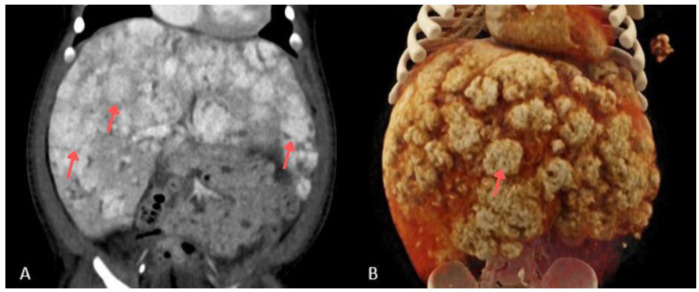
CT of the abdominal cavity with intravenous contrast administration in the venous phase, coronal section (**A**), and CT with Volume Rendering Technique (VRT) (**B**), performed during the first week of hospitalization. Some lesions were marked with arrows.

A 7-week-old infant, the firstborn of a mother with an uncomplicated pregnancy, was admitted for hepatomegaly, non-progressive skin hemangiomas, and anemia requiring transfusion. On physical examination, the infant appeared lethargic and irritable, with a markedly distended abdomen extending above the level of the chest. The patient’s alpha-fetoprotein (AFP) level was 7214.31 ng/mL. The infant experienced a cardiac and respiratory arrest, accompanied by an increase in abdominal circumference, in the context of the overall clinical picture. The infant was intubated and transferred to the paediatric intensive care unit (PICU). A CT scan of the head, chest, and abdomen revealed parenchymal nodular thickening in the lower lung lobes and along the posterior surface of segment 2 of the right lobe and segments 1–2 of the left lobe, as well as numerous lymph nodes in both axillary regions (Figure 1). The liver measured 10.4 cm craniocaudally in the midclavicular line. It showed multiple hypodense round lesions up to 3.3 cm in diameter, along with free fluid in the peritoneal cavity, raising suspicion of multiple hepatic hemangiomas (Figure 2). Propranolol and methylprednisolone were initiated as the first line of treatment. Microbial cultures from tracheal aspirates revealed *Klebsiella pneumoniae*, *Acinetobacter baumannii,* coagulase-negative *Staphylococcus* spp., and *Candida albicans*. Antibiotic therapy with meropenem was introduced. Due to recurrent bacterial lung infections during his hospitalization in the PICU, the patient was administered antifungal prophylaxis with fluconazole at a dose of 10 mg/kg. Two weeks later, *Stenotrophomonas maltophilia* was identified in tracheal aspirates. Due to inadequate response, with only a small reduction in hemangioma size, the propranolol dose was increased (20 mg per day), and sirolimus (with doses modified based on serum concentration) was added to the regimen. On day 52 of hospitalization the patient developed a left-sided pneumothorax, requiring drainage in the course of *Klebsiella pneumoniae* pneumonia. Three days later, after a slight clinical improvement and further small reductions in hemangioma and liver size, respiratory function deteriorated, characterized by tachypnea and increased respiratory effort. Tracheal aspirates revealed *Klebsiella pneumoniae* and *Citrobacter youngae* (AmpC+), sensitive only to imipenem, meropenem, and amikacin. Meropenem was added to the antimicrobial regimen. Nine weeks into hospitalization, the patient was transferred from the PICU to the oncology ward, continuing methylprednisolone at 1 mg/kg/day and sirolimus at therapeutic levels (8–10 ng/mL).

**Figure 3:**
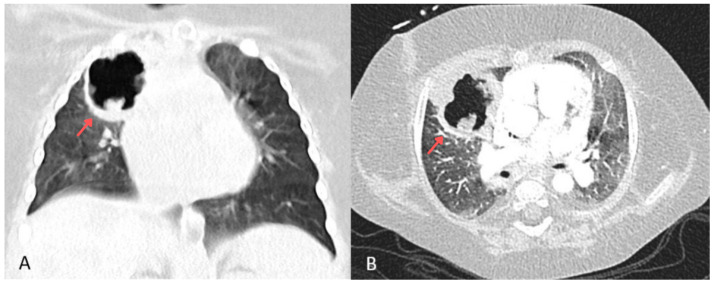
Contrast-enhanced chest CT, coronal section (**A**) and transverse section (**B**), performed 5.5 months after the initial examination. Arrows demonstrate a cavitary lesion in the right upper lung lobe.

**Figure 4:**
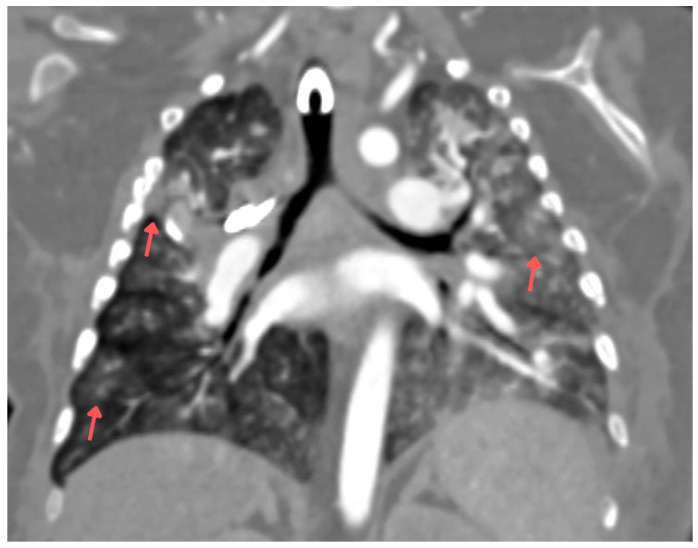
Contrast-enhanced chest CT (coronal section) performed 6 months after the initial examination (10 days prior to death). Some lesions were marked with arrows.

**Figure 5:**
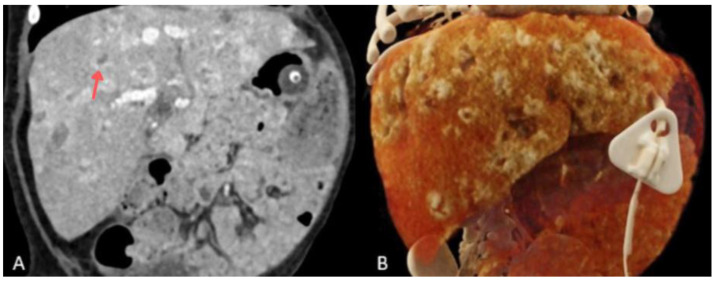
CT of the abdominal cavity with intravenous contrast in the venous phase, coronal section (**A**), and CT with Volume Rendering Technique (VRT) (**B**), performed 6 months after the initial examination (10 days prior to death). One of the lesions marked with an arrow.

**Figure 6:**
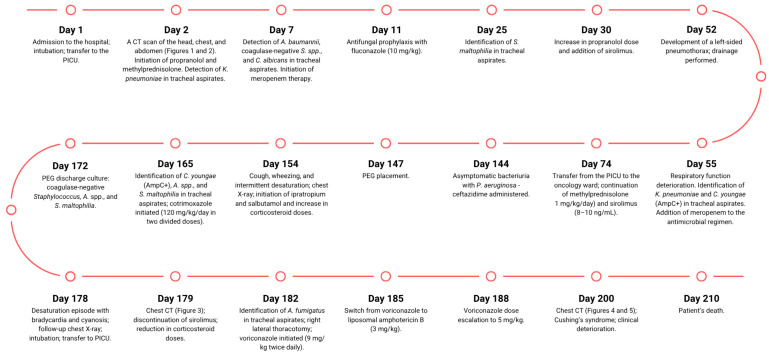
Timeline organizing the most important subsequent events of the patient’s medical history.

After 4.5 months, asymptomatic bacteriuria with *Pseudomonas aeruginosa* was detected and treated with ceftazidime. Due to feeding difficulties, a percutaneous gastrostomy (PEG) was placed. A week later, the patient developed coughing, wheezing, and periodic desaturation episodes, prompting the introduction of ipratropium and salbutamol and increased corticosteroid doses. A chest X-ray revealed worsening parenchymal thickening in both lungs. Tracheal aspirates cultured *Citrobacter youngae* (AmpC+), *Acinetobacter* spp., and *Stenotrophomonas maltophilia*. Intravenous cotrimoxazole was introduced at 120 mg/kg/day in two divided doses. A week later, purulent discharge from the PEG site was cultured, showing coagulase-negative *Staphylococcus*, *Acinetobacter* spp., and *Stenotrophomonas maltophilia*. A week later, the patient experienced a desaturation episode with bradycardia and cyanosis, improving with passive ventilation. No fever, elevation of CRP, and procalcitonin level were observed. A follow-up chest X-ray revealed a necrotic cavitary lesion. Despite changing the tracheostomy tube, respiratory effort worsened, prompting intubation and transfer back to the PICU. Blood testing for galactomannan was negative. Chest CT revealed a cavitary lesion in the right upper lobe with necrotic tissue and airway compression (Figure 3). Sirolimus was discontinued, and corticosteroid doses were reduced. *Aspergillus fumigatus*, sensitive to voriconazole (MIC 0.125) and amphotericin B (MIC determined from two cultures: 0.064 and 0.094), and resistant to caspofungin, was cultured from tracheal aspirates only after the aspergilloma rupture. A right lateral thoracotomy was performed to remove necrotic lung tissue, and *Aspergillus fumigatus* was confirmed in the excised tissue. Voriconazole was initially started at 9 mg/kg twice daily and then switched to liposomal amphotericin B (3 mg/kg) after three days due to insufficient therapeutic concentrations—0.5 mg/L (therapeutic concentration range: 2–5.5 mg/L) with the dose escalation to 5 mg/kg. Despite treatment, lung function did not improve over the next four weeks. A chest CT performed 6 months after the initial examination (10 days before death) showed parenchymal consolidations and a 14 mm × 6 mm subpleural lesion in the right middle lobe, along with scattered ground-glass opacities and septal thickening. The left lung showed diffuse, confluent ground-glass opacities in both lobes (Figure 4). The liver measured 9.1 cm craniocaudally in the midclavicular line bilaterally, which is smaller than previously noted. It appeared heterogeneous and contained multiple round and oval lesions up to 2.0 cm in diameter (Figure 5). Coagulopathy and multiple organ failure progressed, complicated by Cushing’s syndrome secondary to prolonged steroid therapy. The patient died at 9 months of age. Figure 6 shows the timeline of the patient’s medical history.

## 3. Discussion

The first-line treatment for patients with IHH is propranolol, which reduces the growth of hemangiomas and, consequently, improves associated organ failure and hypothyroidism [3]. In severe cases with poor response to propranolol, additional corticosteroid therapy, which significantly contributes to immune suppression, might be considered. Although their use in many patients was never FDA-approved, they remained a predominant treatment for infantile hemangiomas due to their antiangiogenic properties. However, due to their serious long-term side effects, such as growth delay, respiratory complications, gastrointestinal discomfort, adrenal dysfunction, immunosuppression, and hypertension, they have largely been replaced by propranolol [7].

In cases where diffuse hemangiomas persist, treatment with sirolimus, mTOR inhibitor with both antitumor and antiangiogenic properties, may be considered [6]. The treatment for our patient consisted of propranolol, corticosteroids, and sirolimus. The combination of corticosteroids and sirolimus suppresses the immune response, thereby increasing the risk of severe pulmonary complications and the subsequent development of pulmonary aspergillosis.

A markedly enlarged liver can compress the diaphragm, restricting lung expansion and contributing to atelectasis, which, in turn, heightens the risk of respiratory infections. Upon admission, the patient exhibited inflammatory-atelectatic changes perihilar in both lungs and retrocardiac in the lower field of the left lung, likely caused by hepatomegaly. Despite the treatment and reduction in the lesion’s size, the liver remained significantly enlarged, which was correlated with the progression of lung infections. In summary, the combination of immunosuppressive therapy with steroids and sirolimus, impaired clearance of bronchial secretions associated with hepatomegaly, and exposure to hospital-acquired pathogens in the ICU, rendered the patient highly susceptible to infections with virulent pathogens, including fungi.

According to the classification of patients into risk groups for developing invasive fungal disease (IFD) presented at the 10th European Conference on Infections in Leukaemia (ECIL-10), our patient belonged to the sporadic occurrence group for IFD, as a patient with malignancy (other than those in the high- and low-risk groups) [8]. ECIL-10 identifies several factors that elevate the risk of IFD, including prolonged and profound granulocytopenia lasting more than 10 days, recent corticosteroid therapy at a therapeutic dose of ≥0.3 mg/kg for at least 3 weeks within the past 60 days, immunosuppressive treatment within the last 90 days and intensive care unit (ICU) admission. Although the patient was categorized in the sporadic occurrence group for IFD (low-risk group, i.e., risk up to 5%), abnormalities in lung ventilation, along with immunosuppressive therapy, corticosteroid and sirolimus therapy, liver secondary dysfunction due to the presented lesions, central venous catheters (CVC), parenteral nutrition, patient’s age (i.e., infants) and PICU admission, increased her susceptibility to fungal infection [9]. According to ECIL, antifungal prophylaxis is recommended in patients at high risk of IFD, defined as a risk of at least 10%. This high-risk group includes recipients of allogeneic hematopoietic stem cell transplantation and patients diagnosed with acute lymphoblastic leukemia, acute myeloid leukemia, severe aplastic anemia, myelodysplastic syndrome, chronic granulomatous disease, and severe combined immunodeficiency (SCID). In such patients, monitoring for *Aspergillus* spp. is recommended twice weekly using galactomannan testing, particularly in the absence of mold-active antifungal prophylaxis. We consider that this type of monitoring could also potentially be applied to patients in whom the treating physician is concerned about the development of IFD based on the presence of lesser risk factors. For patients at high risk, prophylaxis with posaconazole, which has activity against both yeasts and molds, is recommended. Fluconazole is active only against yeasts and is recommended for use exclusively in centers where the incidence of mold infections is low, and only when diagnostic and therapeutic standards accounting for mold fungi in suspected fungal infections are maintained.

Systemic antifungal prophylaxis is generally contraindicated in patients with malignancies at low risk for IFD, as its benefits are often outweighed by the risks of adverse effects, costs, and inconvenience. Therefore, pediatric patients with solid tumors generally do not require antifungal prophylaxis [10]. However, due to the steroid therapy, the patient received antifungal primary prophylaxis with fluconazole. Nevertheless, it did not prevent the development of pulmonary aspergillosis, as fluconazole’s spectrum of activity does not cover molds due to the inherent and acquired genetic characteristics of *Aspergillus fumigatus* (including mutations in the target enzyme CYP51A and efflux pumps) [11] and is primarily used for the prevention and first-line treatment of candidiasis.

Despite classifying patients with hematologic malignancies according to ECIL as solid tumor patients at low risk for IFD, if at least three of the following risk factors are present: neutropenia (<500/µL, particularly lasting >10 days); recent corticosteroid therapy at a therapeutic dose of ≥0.3 mg/kg for at least 3 weeks within the past 60 days, immunosuppressive treatment within the last 90 days, and PICU admission; mechanical ventilation; CVC; total parenteral nutrition; surgical procedures (especially abdominal surgery); renal failure (particularly requiring dialysis); liver disease; prior colonization with fungi (e.g., in the respiratory or gastrointestinal tract); or neonatal age, the antifungal prophylaxis strategy should be reconsidered depending on the local epidemiological situation, with the potential selection of an alternative antifungal agent other than standard fluconazole.

According to the recommendations for targeted treatment of invasive aspergillosis presented at the ECIL-10, the first-line treatment against *Aspergillus* spp. is voriconazole, with a recommendation grading of A-IIt. In cases of azole resistance, the administration of liposomal amphotericin B is recommended. The recommendation grading for amphotericin B is B-IIt; however, the pivotal phase III trial compared two different dosage strategies without a direct head-to-head comparison to voriconazole at the time of its conduct. Due to the suspicion of the secondary adrenal gland insufficiency after long-term steroid therapy and voriconazole subtherapeutic concentrations, it was discontinued after a short time (three days) and was switched to amphotericin B monotherapy in our patient, given the susceptibility of *Aspergillus fumigatus* to this agent. Despite ongoing advancements in antifungal prophylaxis and treatment methods, invasive aspergillosis remains a highly fatal infection, with historical mortality rates ranging from 52.5% to 85% in children with neoplasms [12].

After the fungal pneumonitis had been suspected according to the chest CT image, the patient was administered voriconazole. However, due to unpredictable voriconazole pharmacokinetics and pharmacodynamics in children, especially those under 24 months of age, the therapeutic concentration of voriconazole was not achieved. The difficulty in attaining the drug’s therapeutic concentration may be attributed to infants’ faster weight-normalized clearance rate and particularly high intra- and inter-individual variability in exposure [13,14]. Although the therapy was switched to liposomal amphotericin B, it did not result in any significant improvement in lung function.

## 4. Conclusions

In conclusion, we report a case of an infant patient with diffuse hepatic hemangiomas leading to recurring severe respiratory and pulmonary infections, complicated by a cavitary lesion with necrotic tissue due to aspergillosis and airway compression. This case highlights the complexity of managing infantile hepatic hemangiomas. The treatment regimen of corticosteroids and sirolimus contributed to immunosuppression, likely increasing the patient’s susceptibility to infections, including recurrent lung infections and treatment-resistant pulmonary aspergillosis. The substantial hepatomegaly further compounded the issue by causing diaphragm compression and atelectasis, which worsened respiratory function and predisposed the patient to infections. Despite aggressive treatment, the combination of immunosuppressive therapy and organ complications led to a poor outcome, underscoring the need for careful monitoring and individualized treatment in such complex cases.

## Data Availability

Data is unavailable due to patients’ privacy and ethical restrictions.

## Abbreviations

The following abbreviations are used in this manuscript:

IHH	Infantile hepatic hemangiomas
IH	Infantile hemangiomas
DHH	Diffuse hepatic hemangiomas
AFP	Alpha-fetoprotein
PICU	Paediatric intensive care unit
VRT	Volume Rendering Technique
PEG	Percutaneous gastrostomy
IFD	Invasive fungal disease
ECIL-10	10th European Conference on Infections in Leukaemia
ICU	Intensive care unit
